# Work-Related Stressors among the Healthcare Professionals in the Fever Clinic Centers for Individuals with Symptoms of COVID-19

**DOI:** 10.3390/healthcare9050548

**Published:** 2021-05-08

**Authors:** Saad Alyahya, Fouad AboGazalah

**Affiliations:** 1 Nudge Unit, Ministry of Health, Riyadh 12628, Saudi Arabia; 2 Family Medicine, Health Holding Company, Riyadh 12584, Saudi Arabia; Fabogazalah@moh.gov.sa

**Keywords:** stress, role conflict, role ambiguity, social support, COVID-19, Saudi Arabia

## Abstract

Work-related stress can affect the quality of healthcare during the COVID-19 pandemic. This study aimed to assess the relationship between selected work-related stressors and stress levels among healthcare professionals providing preventive and curative services to people with COVID-19 symptoms in the Fever Clinics in Saudi Arabia. A systematic random sampling using an online questionnaire approach was used to select healthcare professionals in the Fever Clinics in Saudi Arabia during September 2020. Participants were asked to fill out a questionnaire including data on their sociodemographic and occupational characteristics, role conflict and ambiguity, social support, and stress. The results showed that role conflict and ambiguity were significant risk factors for stress, and social support was negatively associated with stress levels. Additionally, younger and non-Saudi healthcare professionals exhibited higher stress levels than their older and Saudi counterparts. In conclusion, role conflict, ambiguity, and social support can predict the risk of stress among healthcare professionals in the Fever Clinics in Saudi Arabia.

## 1. Introduction

Before the end of 2019, the World Health Organization (WHO) was informed of pneumonia cases discovered in Wuhan, Hubei Province, China, which was named the 2019 novel coronavirus disease (COVID-19) caused by the novel coronavirus (SARS-CoV-2) [1].

On 11 March 2020, the World Health Organization declared coronavirus (COVID-19) a pandemic, which means a global disease outbreak threatening the world [2,3] After the World Health Organization announced the pandemic, Saudi Arabia adopted response plans to contain the virus [4]. From 2 March 2020, to 2 March 2021, Saudi Arabia confirmed 377,700 cases of COVID-19 with 6500 deaths [5].

The Kingdom of Saudi Arabia has implemented many precautionary measures to prevent the spread and transmission of the SARS-CoV-2 virus, such as preventing mass gatherings, stopping school proceedings, stopping Hajj and Umrah, introducing curfews, and closing places to counter the increase in the transmission of infection [6,7,8]. The country has converted more than 119 health centers to receive only those infected with infection symptoms; they cover all regions and cities of the Kingdom of Saudi Arabia and receive citizens, residents, and immigrants into the country. These clinics, known as Fever Clinics, work to contain the coronavirus’s spread among society members [9].

COVID-19 is the world’s biggest public health threat. The severity of the disease, the lack of evidence of the effectiveness of potential therapeutic agents and the available vaccines, and the lack of presumed immunity in the population leave everyone vulnerable to infection [10,11]. Additionally, the rapid spread of COVID-19, its health implications, the wide coverage that this disease gets in the press and social media, and the horrific statistics associated with it may lead to an increase in anxiety levels and the level of adverse effects on mental health among members of society [12,13].

A number of members in the society have been affected by different psychological influences, including health care workers, who are considered one of the essential elements in protecting community members from having the virus [12,14,15]. Several studies revealed that fear of this novel coronavirus might lead to psychological issues such as stress disorders, anxiety, and depression among individuals [16]. This outbreak is not the first in the Kingdom of Saudi Arabia, since it has experienced the MERS virus epidemic before. This epidemic spread to the Middle East, affecting health workers and killing some of them, causing many psychological problems such as stress [17,18,19].

Stress may be affected by many variables related to the professional and social role, such as ambiguity, role conflict, and increased workload [20]. Several studies have found that workload, role ambiguity, and role conflict are factors associated with stress and that these factors degrade employee job satisfaction and performance [21,22,23]. Based on this, too much stress may negatively affect both the employees’ and the organization’s work performance.

In contrast, social support is a preventative factor against mental health problems in the workplace [24]. Social support is the care or help that a person can feel, notice, or accept from others. A high social support level can protect someone under stress [25,26]. The more social support the person has or perceives, the more control of the person’s stressful situation, leading to improved outcomes associated with it [27,28].

All these factors, including role ambiguity, role conflict, work overload, and social support, may influence the level of stress among health sector workers, especially during the novel coronavirus pandemic.

Therefore, to the authors’ knowledge, this study is considered the first of its kind. This study seeks to determine the relationship between the variables associated with stress, such as ambiguity and role conflict, workload, and social support, among health sector workers, specifically in health centers called Fever Clinics centers designated to receive those with symptoms of COVID-19 infection in the Kingdom of Saudi Arabia.

## 2. Materials and Methods

This study used data from Almansour et al., study of Work-Related Challenges among Primary Health Centers Workers during COVID-19 in Saudi Arabia [29]. The random sampling technique was used in the original national cross-sectional study. The original study aimed to explore the association between role conflict, role ambiguity, self-esteem, and social support with stress levels among HCWs in primary healthcare centers in Saudi Arabia, including regular primary health centers and Fever Clinic centers. The original study also aimed to identify the differences in stress, role conflict and ambiguity, self-esteem, and social support between employees in regular healthcare centers and Fever Clinics.

An online questionnaire was used to collect data from the participants using a multistage random sampling approach. The study participants were selected from the employees in the primary healthcare center of the five geographical regions of Saudi Arabia. The study participants were recruited from 20 Directorates of Health Affairs in Saudi Arabia divided into Central, Eastern, Western, Northern, and Southern regions. All the public government primary health care centers (regular or Fever Clinic centers) fall in these 20 Directorates of Health Affairs, so every primary health care center has a chance to be selected in this study. The number of regular primary healthcare centers was 2070, while the number of Fever Clinic centers was 119. In the original study, six regular health centers and two Fever Clinics centers were randomly selected from directorates in all regions. An email was sent to each employee in the selected health centers, and a reminder email was sent a week later. A link to an electronic self-administrated questionnaire was provided in the email, and it was anticipated to be completed in 10 min. The time frame for data collection was three weeks began on 27 September 2020.

The design of the present study was nonexperimental, correlational, and cross-sectional. This study aimed to understand the sample characteristics better and investigate the work-related factors (conflict, ambiguity, overload, and social support) that can contribute to stress among health care workers in the Fever Clinic centers.

### 2.1. Sample

The original study’s target population was the various employees in the primary healthcare centers, including healthcare professionals such as nurses, physicians, pharmacists, social workers, and the administration department employees in the healthcare centers. Emails were sent inviting the employees from the randomly selected healthcare centers to take part in the study. The emails sent out saw a 69% response rate: 1378 responses were received from the 2007 emails sent out. The data collection in each health directorate was coordinated by the head of the primary healthcare center Health Affairs Directorate. The link to the self-administered electronic questionnaire was mailed to the employees by the head of the primary healthcare center. The participants were assisted by the principal investigator when they experienced any challenges with the questionnaire. The target participants also received a weekly reminder during the data collection period.

This current study has a sample size *N* = 275 of health care workers extracted from the original dataset. The current study selected only health care workers who work in the Fever Clinics centers (20% of the original sample); health care workers who work in the regular health care centers were excluded from the current study (80% of the original sample). A conducted power analysis of the currently selected sample size of *N* = 275 using G*Power software, version 3.1.9.7, showed that this sample size has around 80% power (1-Beta error) at an alpha of 0.05, with four predictors, and one criterion, to detect even a modest effect size of 0.04 [30].

### 2.2. Study Variables

The study had both dependent and independent variables. The study’s dependent variable was stress, while there were several independent variables, including work conflict, work ambiguity, work overload, and social support.

#### 2.2.1. Stress

Lazarus and Folkman defined stress as “a relationship with the environment that the person appraises as significant for his or her wellbeing and in which the demands tax or exceed available coping resources” [20]. Stress was measured using the Perceived Stress Scale (PSS) about events in the last month using the following items: upset because of something that happened unexpectedly; unable to control the important things in your life; nervous and “stressed”; confident about your ability to handle your personal problems; things were going your way; you could not cope with all the things that you had to do; you have been able to control irritations in your life; you were on top of things; angered because of things that were outside of your control; and difficulties were piling up so high that you could not overcome them. This scale has been used significantly in measuring the stress estimates with strong psychometric properties [31]. Cohen et al. argue that PSS is highly correlated with various scales, making it have good validity. These may include health behavior measures, smoking status, help-seeking behavior, self-reported health, and health services measures. The participants assessed the intensity to which life stressors were overwhelming and unmanageable over the previous month. They used a scale ranging from 0 (never) to 4 (very often), with a higher score referring to a higher stress level. The scale in the original study has good reliability and validity (Cronbach’s alpha 0.85).

#### 2.2.2. Work Role Conflict

Role conflict is “the extent to which one experiences incompatible work demands” [32]. This study uses the role conflict measure for workers developed by Bowling et al. to evaluate the work-role conflict among primary healthcare workers [32]. The measure has six items and provided answers ranging from 1 (strongly disagree) to 7 (strongly agree), with a higher score on the scale pointing to a higher conflict level. The scale includes the following items: in my job, I often feel like different people are “pulling me in different directions”; I have to deal with competing demands at work; my superiors often tell me to do two different things that cannot both be done; the tasks I am assigned at work rarely come into conflict with each other; the things I am told to do at work do not conflict with each other, in my job; and I am seldom placed in a situation where one job duty conflicts with other job duties. In the original study, the scale has a reliability (Chronbach’s Alpha = 0.62) which is acceptable reliability [33], as well as criterion validity with the Perceived Stress Scale (r = 0.472, *p* < 0.01).

#### 2.2.3. Work Role Ambiguity

This variable is defined as “The extent to which one is confronted with unclear work situations” [32]. The ambiguity instrument used in this study measures role ambiguity using six items measured on a 7-point Likert scale ranging from 1 (strongly disagree) to 7 (strongly agree). The scale includes the following items: I am not sure what is expected of me at work; the requirements of my job are not always clear; I often do not know what is expected of me at work; I know everything that I am expected to do at work with certainty; My job duties are clearly defined; and I know what I am required to do for most or every aspect of my job. A higher score on this scale reflects higher role ambiguity. In the original study, the scale has good reliability (Chronbach’s Alpha = 0.85) and a suitable criterion validity with Perceived Stress Scale (r = 0.478, *p* < 0.01).

#### 2.2.4. Work Overload

Work overload has been defined as the extent to which the “job performance required in a job is excessive or overload due to performance required on a job” [34]. In the original study, work overload is measured using one question. The participants were asked how many hours they work every day. Their answers are the number of working hours.

#### 2.2.5. Social Support

The Multidimensional Scale of Perceived Social Support (MSPSS) was used for this variable. MSPSS identifies perceptions of support from three critical elements of individuals’ emotional support: family, friends, and significant others; and has 12 self-administered items [35]. The scale items are: there is a special person who is around when I am in need; there is a special person with whom I can share joys and sorrows; my family really tries to help me; I get the emotional help and support I need from my family; I have a special person who is a real source of comfort to me; my friends really try to help me; I can count on my friends when things go wrong; I can talk about my problems with my family; I have friends with whom I can share my joys and sorrows; there is a special person in my life who cares about my feelings; my family is willing to help me make decisions; and I can talk about my problems with my friends. The answer choices are in the form of a 7-point Likert scale ranging from 1 (very strongly disagree) to 7 (very strongly agree), with a higher score on the scale showing higher social support. Original study found that the scale had good reliability (Cronbach’s alpha 0.92) and criterion validity (r = −0.332, *p* < 0.01).

### 2.3. Data Analysis

This study provided descriptive analysis, including measures of central tendency and variability for the demographic characteristics. Additionally, the chi-square test was used to compare the participants’ characteristics based on the stress levels (low, moderate, and high). Bivariate correlation examined individual relationships between the level of stress (criterion variable) and the predictors. Multiple regression analysis was used to identify which of the work-related factors were best predictors for stress level. Beta scores (standardized coefficients) allowed identifying those variables with the most significant contribution to informal caregivers’ emotional stress.

## 3. Results

Table 1 provides the descriptive statistics for the demographic variables to give the respondents’ background characteristics. The average age of respondents is 38.9 years, with a standard deviation of 8.12. The results show that 36 (13.1%) respondents are less than 30 years of age, 186 (67.8%) are between ages 31 and 45, and 53 (19.3%) are 46 or older. For gender, the percentage of male respondents tends to be higher than that of female, which is 154 (56%) and 121 (44%), respectively. Additionally, most of the respondents (84.7%) are married or living together, 9.5% have never been married or single, while only 3.6% had earlier been married but then divorced or separated. The great majority of the respondents, 230 (83.6%), are citizens, while only 45 (16.4%) of them are non-residents or non-citizens. Regarding their education level, most of the respondents have attained two years of college education (42.6%), followed by those with bachelor’s education (36.7%), 9.5% in graduate-level, and 7.6% attaining high school education or less. Regarding the experience with primary health centers (PHCs), the majority are those with more than ten years of experience (48.4%), 28.7% of the respondents have five years or less of experience, and those with 6 to 10 years of experience are only 22.9% of the respondents.

Table 2 shows the Pearson Correlation coefficients between the study’s independent variables (conflict, ambiguity, overload, and social support) and the dependent variable of stress. All the independent variables in the study have a positive significant correlation with stress except overload, which has a significant negative correlation. The result was: conflict (*r* = 0.487, *p* < 0.01), ambiguity (*r* = 0.479, *p* < 0.01), overload (*r* = −0.332, *p* < 0.01), and social support (*r* = 0.090, *p* < 0.01). Additionally, conflict was found significantly correlated with ambiguity (*r* = 0.509, *p* < 0.01), overload (*r* = −0.206, *p* < 0.01), and social support (*r* = 0.079, *p* < 0.01). Ambiguity exhibited significant negative correlation with overload (*r* = −0.265, *p* < 0.01) and social support (*r* = −0.026, *p* < 0.01). Finally, overload had a negative significant correlation with social support (*r* = −0.013, *p* < 0.01). These results give a clear evidence that most of the independent variables exhibited significant correlation with each other.

The study used a chi-square test to compare healthcare workers’ demographics and working hours in the Fever Clinics to improve the understanding of their characteristics based on their stress level (Low, Moderate, and High). These results are presented in Table 3. The result showed a significant difference related to participants’ age groups. Younger health care workers more likely to have higher stress than the other age groups. The analysis on workers’ nationality showed significant differences; non-Saudi health care workers have higher stress than Saudis. Gender, marital status, and level of education did not show significant differences in their level of stress. Additionally, working hours change did not reveal any significant differences in the stress level among the Fever Clinics’ health care workers.

Table 4 shows multiple regression analysis results between the criterion of stress and the predictors of conflict, ambiguity, social support, and overload. The multiple regression results indicated a significant collective effect between the independent variables of conflict, ambiguity, social support, and the dependent variable of stress. The overload was dropped from the model by the SPSS and it might be dropped because of using a single item to measure the overload. According to the result, the significant predictors based on their magnitude are: (ambiguity (*Beta* = 327, *p* < 0.001), conflict (*Beta* = 313, *p* < 0.001), and social support (*Beta* = −0.150, *p* < 0.01)). R square = 0.36, so all of these factors together explained 36.4% of perceived stress.

## 4. Discussion

This study unveiled several work-related risk factors for stress among healthcare professionals managing COVID-19 in the Fever Clinics in Saudi Arabia. While role conflict and ambiguity were positively associated with stress levels, social support was inversely associated with stress levels.

Our results concurred with a recent study conducted on healthcare professionals in primary healthcare centers in Saudi Arabia during the COVID-19 time that showed high role conflict and ambiguity levels, especially among those working at the Fever Clinics. This research also showed a tight correlation between work-related stressors and stress levels [29]. Additionally, one study in the pre-COVID-19 time conducted on healthcare professionals from Italy showed that role ambiguity was a significant risk factor for emotional exhaustion and negatively affected participants’ wellbeing and psychosocial competence [36].

The Minister of Health in Saudi Arabia initiated the Fever Clinics in the wake of the COVID-19 pandemic to offer preventive and curative services for people showing COVID-19 symptoms. Given the rapidly changing guidelines and protocols of COVID-19 management and the fact that the scientific knowledge on the virus is still limited, the Fever Clinics’ healthcare professionals might have incomplete details on their job requirements. Together, these factors can explain role conflict and ambiguity and the resulting stress among health care professionals working at the Fever Clinics [29]

The inverse association between social support and stress levels was expected. Two recent studies conducted on healthcare professional in Saudi Arabia showed that, during the COVID-19 pandemic, participants who did not perceive enough emotional support from society and the workplace had higher stress levels than those who perceived enough emotional support [29,37]. This finding elucidates the need to provide social support to healthcare professionals on the frontlines to improve their psychological health.

Of note, this study also showed that younger and non-Saudi healthcare professionals were more likely to report stress than their older and Saudi counterparts. The vulnerability of younger people to stress during the COVID-19 pandemic has been reported, highlighting the importance of tailoring mental health interventions for this age group [38]. The disparity in stress levels between Saudi and non-Saudi healthcare professionals can be attributed to the fact that non-Saudi healthcare professionals could not travel to their home countries because of the travel restrictions associated with the COVID-19 pandemic. It could also reflect a worse occupational environment or fewer financial motivations. However, this association should be further studied in future research.

## 5. Strengths and Limitations

This study had many strengths, including the multistage random sampling approach, using validated and reliable scales data to collect data on the work-related stressors and stress levels, and focusing on healthcare professionals in the frontlines managing COVID-19. Still, some limitations should be addressed. First, since the cross-sectional design cannot guarantee causality, studies with prospective designs are needed to confirm our results. Second, the data collecting tool had no information on the lack of personal protective equipment, one of the significant stressors among healthcare professionals during the COVID-19 time. Third, we accessed participants using emails. Online surveys have many advantages such as cutting time, saving funds, decreasing missing data, yet they include some limitations such as the high possibility of non-response bias. Since we have no data on the nonrespondents, we cannot guarantee that the respondents had similar sociodemographic data and work-related stressors [39].

## 6. Conclusions

The current study showed that among healthcare professionals working at the Fever Clinics and managing COVID-19 in Saudi Arabia, role conflict and ambiguity were positively associated with stress levels. In contrast, social support was inversely associated with social support. We believe that the reasons behind role conflict and ambiguity among healthcare professionals in the Fever Clinics in Saudi Arabia should be studied, and tailored interventions should be put into practice.

## Figures and Tables

**Table 1:** Demographic characteristics.

Variable	*n*	%	M	SD
**Age**			38.9	8.13
Less than 30 years	36	13.1		
31 to 45 years	186	67.6		
46 years or older	53	19.3		
**Gender**				
Male	154	56.0		
Female	121	44.0		
**Marital Status**				
Single	32	9.5		
Married	233	84.7		
Divorced	10	3.6		
**Nationality**				
Citizen	230	83.6		
Not citizen	45	16.4		
**Education Level**				
High school or less	21	7.6		
Two years college	127	46.2		
Bachelor	101	36.7		
Graduate	26	9.5		
**Experience in PHCs**				
5 years or less	79	28.7		
6–10 years	63	22.9		
More than 10 years	133	48.4		

**Table 2:** Pearson Correlation between stress and the study variables.

Variable	1	2	3	4	5
Stress	1				
Conflict	0.487 **	1			
Ambiguity	0.479 **	0.509 **	1		
Overload	−0.332 **	−0.206 **	−0.265 **	1	
Social support	0.090 **	0.079 **	−0.026 **	−0.013 **	1

Note. ** Correlation is significant at the 0.01 level (2-tailed).

**Table 3:** Demographic characteristics by stress levels.

Variables	Low Stress Mean (±SD)	Moderate Stress Mean (±SD)	High Stress Mean (±SD)	*p*-Value
**Age**				
18–35 years	21 (30.4%)	72 (43.4%)	22 (55%)	*p* < 0.05
36–45 years	29 (42%)	61 (36.7%)	17 (42.5%)	
46–60 years	19 (27.5%)	33 (19.9%)	1 (2.5%)	
**Gender**				
Male	23 (33.3%)	78 (47%)	20 (50%)	*p* > 0.05
Female	46 (66.7%)	88 (53%)	20 (50%)	
**Nationality**				
Saudi	20 (29%)	22 (13.3%)	3 (7.5%)	*p* < 0.01
Non-Saudi	49 (71%)	144 (86.7%)	37 (92.5%)	
**Marital Status**				
Not Married	6 (8.7%)	32 (19.3)	4 (10%)	*p* > 0.05
Married	63 (91.3%)	134 (80.7%)	36 (90%)	
**Education level**				
High school or less	7 (10.1%)	13 (7.8%)	1 (2.5%)	*p* > 0.05
Two years college	32 (46.4%)	76 (45.8%)	19 (47.5)	
Bachelor	23 (33.3%)	64 (38.6%)	14 (35%)	
Graduate	7 (10.1%)	13 (7.8%)	6 (15%)	
**Since COVID-19 work**				
More hours	40 (58%)	112 (67.5%)	26 (65%)	*p* > 0.05
Same or fewer hours	29 (42%)	54 (32.5%)	14 (35%)	

**Table 4:** Multiple regression analysis: predictors of stress.

Variable	B	SE	β	*t*	*p*	95.0% Confidence Interval for B	
						Lower Bound	Upper Bound
Ambiguity	2.409	0.416	0.327	5.794	0.000	1.591	3.228
Conflict	0.336	0.060	0.313	5.559	0.000	0.217	0.455
Social Support	−0.091	0.030	−0.150	−3.035	0.003	−0.149	−0.032
Constant	6.416	2.687		2.388	0.018	1.127	11.706
Adjusted *R*^2^		0.364					
*F*		53.31 **					

*Note.* ** *p* < 0.01 level (2-tailed).

## Data Availability

Data is available upon reasonable request by contacting the corresponding author.
